# Glycemic Control for Colorectal Cancer Survivors Compared to Those without Cancer in the Dutch Primary Care for Type 2 Diabetes: A Prospective Cohort Study

**DOI:** 10.3390/cancers13112767

**Published:** 2021-06-02

**Authors:** Jing de Haan-Du, Gijs W. D. Landman, Nanne Kleefstra, Dennis Schrijnders, Marjolijn Manders, Amanda C. R. K. Bos, Cathrien Tromp-van Driel, Petra Denig, Klaas H. Groenier, Geertruida H. de Bock

**Affiliations:** 1 Department of Epidemiology, University Medical Center Groningen, University of Groningen, 9713 GZ Groningen, The Netherlands; g.landman@gelre.nl (G.W.D.L.); g.h.de.bock@umcg.nl (G.H.d.B.); 2 Langerhans Medical Research Group, 7731 AT Ommen, The Netherlands; nanno@kleefstra.org (N.K.); dschrijnders@gmail.com (D.S.); m.manders@umcg.nl (M.M.); 3 Department of Internal Medicine, Gelre Hospital, 7334 DZ Apeldoorn, The Netherlands; 4 Department of Forensic Psychiatry, GGZ Drenthe Mental Health Institute, 9404 LA Assen, The Netherlands; 5 Department of Internal Medicine, University Medical Center Groningen, University of Groningen, 9713 GZ Groningen, The Netherlands; 6 Department of Research & Development, Netherlands Comprehensive Cancer Organisation (IKNL), 3511 DT Utrecht, The Netherlands; a.bos@iknl.nl; 7 Department of Oncology, Gelre Hospital, 7334 DZ Apeldoorn, The Netherlands; c.tromp@gelre.nl; 8 Department of Clinical Pharmacy and Pharmacology, University Medical Center Groningen, University of Groningen, 9713 GZ Groningen, The Netherlands; p.denig@umcg.nl; 9 University of Groningen, 9713 GZ Groningen, The Netherlands; k.h.groenier@rug.nl

**Keywords:** cancer survivors, colorectal neoplasms, glycated hemoglobin a, primary healthcare, diabetes mellitus, type 2

## Abstract

**Simple Summary:**

A growing number of colorectal cancer survivors live with type 2 diabetes, as a result of improved cancer diagnosis and treatment. These patients might have worse glycemic control after their cancer diagnosis, which may increase the risk of cardiovascular diseases. This prospective cohort study evaluated the quality of glycemic control for colorectal cancer survivors, as compared to those without cancer in Dutch primary care for diabetes. During a 10-year follow-up for 57,330 patients, there were 705 patients diagnosed with colorectal cancer. No clinically relevant difference on the probability of reaching the target HbA1c was observed between colorectal cancer survivors and patients with no history of cancer. These results showed a robust diabetes care system, implying that the glycemic control for colorectal cancer survivors can be delegated to the primary care professionals.

**Abstract:**

Cancer survivors with diabetes tend to have worse glycemic control after their cancer diagnosis, which may increase the risk of cardiovascular diseases. We aimed to investigate whether glycemic control differs between colorectal cancer (CRC) survivors and those without cancer, among patients with type 2 diabetes being treated in the Dutch primary care. The Zwolle Outpatient Diabetes project Integrating Available Care database was linked with the Dutch Cancer Registry (*n* = 71,648, 1998–2014). The cases were those with stage 0–III CRC, and the controls were those without cancer history. The primary and secondary outcomes were the probability of reaching the glycated hemoglobin (HbA1c) target and the mean of HbA1c during follow-up, respectively. Mixed linear modeling was applied, where the status of CRC was a time-varying variable. Among the 57,330 patients included, 705 developed CRC during follow-up. The mean probability of reaching the HbA1c target during follow-up was 73% versus 74% (*p* = 0.157) for CRC survivors versus those without cancer, respectively. The mean HbA1c was 51.1 versus 50.8 mmol/mol (*p* = 0.045) among CRC survivors versus those without cancer, respectively. We observed a clinically comparable glycemic control among the CRC survivors without cancer, indicating that glycemic control for CRC survivors can be delegated to primary care professionals.

## 1. Introduction

Around 20% of colorectal cancer (CRC) survivors also have type 2 diabetes [1,2,3]. Improvement in CRC screening, diagnosis, and treatment has significantly increased the 5-year survival for non-metastatic CRC to 70–91% [4,5,6]. Compounded by an aging society, a growing number of CRC survivors living with type 2 diabetes is expected [7,8,9], which may confer a high necessity for these patients to be followed, in primary care.

More than 50% of CRC survivors over 65-years old were reported to develop cardiovascular diseases (stroke and myocardial infarction), in a 10-year follow-up study [10]. Maintaining good glycemic control for these patients is important because worsening glycemic control is associated with an elevated risk of cardiovascular diseases [11]. Furthermore, a cancer diagnosis may have a negative impact on diabetes management, because oncologists and patients may prioritize cancer treatment over diabetes management. This is because certain cancer treatment such as chemotherapy may influence the quality of glycemic control, and because patients may feel overwhelmed and overburdened when they get a diagnosis of cancer [12,13]. For cancer patients with diabetes, there tends to be a decline in diabetes care after a cancer diagnosis, including self-management behaviors, glucose monitoring and treatment, and medication adherence [14]. Compared with diabetes patients without cancer, cancer patients with diabetes used less diabetes care, such as less HbA1c testing [15]. Previous studies usually compared the trajectory of glycemic control before and after the cancer diagnosis while no control group of patients without cancer was introduced [16]. These studies were also further limited by a small sample size and a short follow-up [17,18,19].

In The Netherlands, more than 90% of patients with type 2 diabetes, including cancer survivors diagnosed with concurrent diabetes, are treated in a primary care system provided by general practitioners and specialized nurses [20,21]. This care system has become the Dutch standard care for diabetes, after showing improved quality of care over years, regardless of age and gender [22,23]. However, whether a good glycemic control among CRC survivors can be maintained by primary care professionals is unknown. This study aimed to evaluate the level of glycemic control in CRC survivors, as compared to those without cancer history in the Dutch primary care system for type 2 diabetes.

## 2. Results

**Figure 1:**
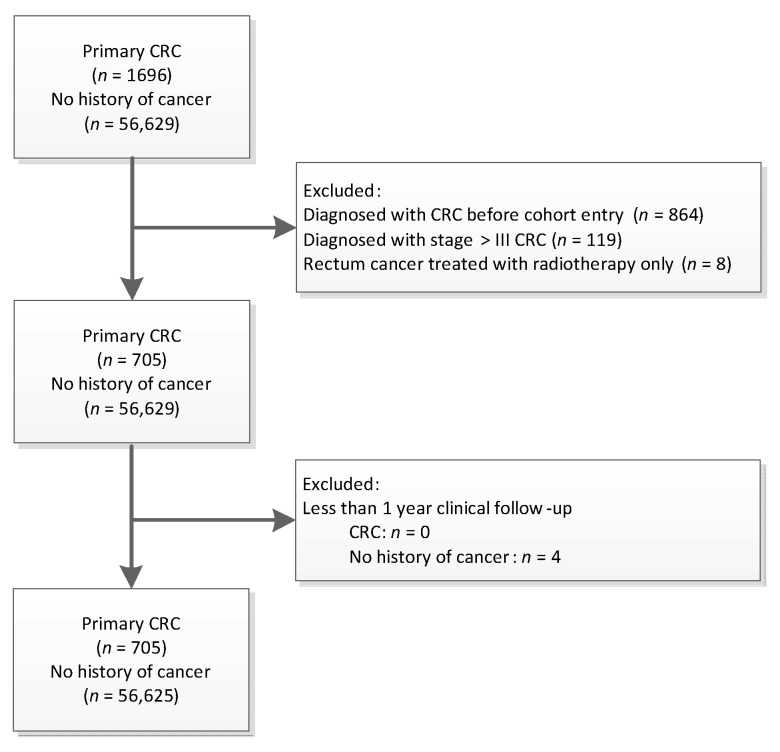
Study flow chart of patients included in the analysis.

Among a total of 57,330 patients (Figure 1), 705 patients diagnosed with CRC were followed for a median of 6 (IQR: 4–8) years and those with no history of any cancer were followed for a median of 5 (IQR: 3–7) years. After cancer diagnosis, the CRC survivors were followed for a median of 2 (IQR: 1–4) years. Table 1 presents the characteristics for all patients at baseline and during follow-up. Patients diagnosed with CRC during follow-up tended to be older, were more likely to be males, and had a slightly longer duration of diabetes when entering the cohort.

Of the total 71,648 patients in ZODIAC and NCR linkage, there were 13,323 patients excluded from the flow-chart as a diagnosis of other type of cancer, except for non-melanoma skin cancer. ZODIAC stands for the Zwolle Outpatient Diabetes project Integrating Available Care and NCR stands for the Dutch National Cancer Registration. The linkage procedure of these two databases was lastly performed in December 2020, in which complete cancer events were observable up to 31 December 2019.

**Figure 2:**
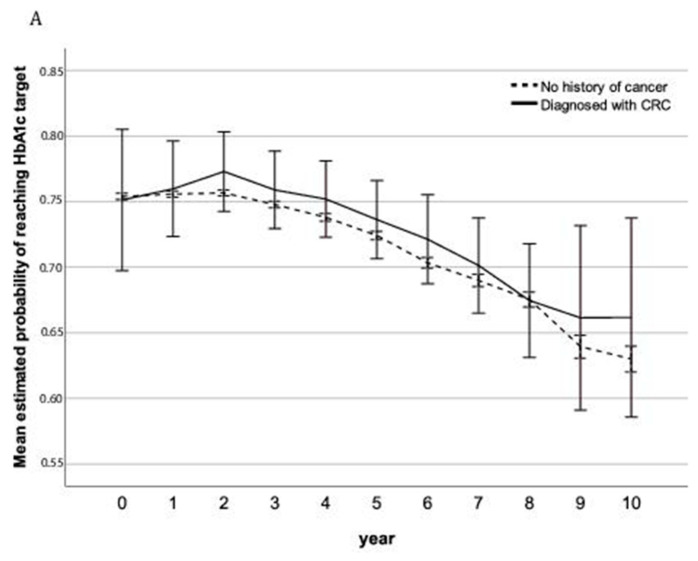

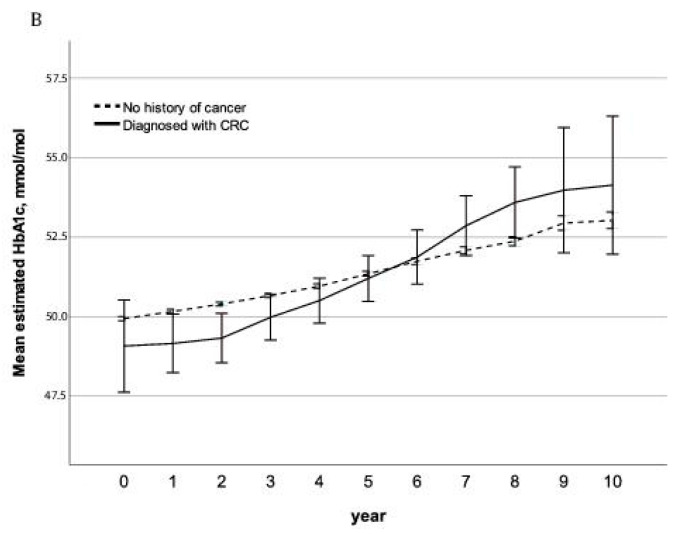
Estimated mean of the outcomes (**A**) Estimated mean probability at target HbA1c level during follow-up. (**B**) Estimated mean HbA1c (mmol/mol) during follow-up. Year 0 represents the cohort entry year, and year 10 represents the data for patients being followed for 10 years. Adjusted for baseline age, gender, diabetes duration, number of oral drugs, use of insulin and baseline year. The horizontal bars represent the 95% confidence intervals.

More than 98% of patients were followed for no longer than 10 years, therefore, the results for 10 follow-up years are presented. The estimated mean probability of reaching the HbA1c target and mean HbA1c in each year are presented in Figure 2 and Table 2, and the detailed regression parameters of the fixed effects in the models are shown in Tables S1 and S2. Overall, the probability of reaching the target HbA1c level decreased during follow-up. The overall mean probability reaching HbA1c target were 73% versus 74% for CRC survivors versus those with no history of cancer. While there was no significant difference in the overall mean probability (*p* = 0.157) as well as the annual change rate of the probability (*p* = 0.260). With regards to the trajectory of mean HbA1c, the overall mean was comparable (51.1 vs. 50.8 mmol/mol, *p* = 0.045). Sensitivity analysis showed the overall mean probability reaching the HbA1c target were 72% versus 74% for CRC survivors versus those with no history of cancer, with a significantly lower overall mean probability (*p* = 0.018) for CRC survivors (Figure S1 and Table S3). The overall mean among patients being followed for at least 5 years was comparable (51.3 vs. 50.6 mmol/mol, *p* = 0.283) (Figure S1 and Table S4).

## 3. Discussion

### 3.1. Summary

Evaluating 10 year follow-up data, CRC survivors with type 2 diabetes being treated in the Dutch primary care achieved comparable quality of glycemic control, as compared to those without a history of cancer. Cancer survivors had a non-significant 1% lower probability reaching the target and non-significant 0.3 mmol/mol higher mean HbA1c, as compared to patients without cancer history. These results indicate that cancer survivors can be treated by primary care professionals without relevant decreases in quality of glycemic control, a proxy for quality of diabetes care.

### 3.2. Comparison with Literature

There were four studies evaluated on the quality of glycemic control among CRC patients. One evaluated the proportion of time to reach the HbA1c target among CRC survivors as compared to patients without cancer in the British primary care setting [24], and three investigated the trend of mean HbA1c before and after cancer diagnosis from the perspective of cancer patients alone [17,18,19]. In the British study, CRC survivors with diabetes showed a 12% lower proportion of time-period in reaching a target HbA1c, as compared to controls without cancer [24]. These results seem different as compared to the current study, in which no differences on the probability of reaching the target HbA1c value in each follow-up year were found. This difference could be explained by differences in quality of diabetes care in different countries [25] and by that the British study evaluated the care quality between 2003–2006 [24], while 78.5% of the patients in the ZODIAC cohort were enrolled after 2006 [22,23]. It could also be possible that a difference in the definition of quality of diabetes care, by incorporating the age- and diabetes-duration-adjusted target HbA1c values since 2013, has partly resulted in this difference. CRC survivors in our study were more prone to reach the adjusted target, as they tend to be older and had longer duration of diabetes as compared to patients with no history of cancer [26]. This explanation is consistent with the results of our sensitivity analysis for patients followed for at least 5 years, where a lower overall mean of the probability of reaching the HbA1c target with a comparable overall mean HbA1c were shown. These patients entered the cohort in 2010 at the latest, when the adjusted HbA1c target were not applied.

The three studies that focused on the mean HbA1c did not have a control group of patients with diabetes and without cancer history, and the trends of mean HbA1c trajectory among CRC patients alone were evaluated [17,18,19]. One study showed 1 mmol/mol per year increase of HbA1c among patients with only colon cancer but not rectum cancer [17], one showed no significant change [18], while the third showed a decline on the mean HbA1c [19]. These three studies were limited to only 1- or 2-years follow-up after cancer diagnosis [17,18] and a small sample size (*n* = 85) of only 55% of HbA1c information was available [19]. In our study in the Dutch primary care, an increasing trend was shown in the mean HbA1c among CRC survivors, but this was not clinically relevant and less than 1 mmol/mol increase per year was observed. Again, this suggests that CRC survivors similarly benefit from the high quality of care, as other patients with type 2 diabetes.

### 3.3. Strengths and Limitations

This study has several strengths. Instead of investigating the quality of glycemic control from the perspective of cancer patients alone, data collected from the Dutch primary care system offers a unique perspective to evaluate the quality of glycemic control for cancer survivors as compared with no cancer patients. The prospectively collected clinical data from 1998 until 2014 allowed us to evaluate the quality of glycemic control with a long follow-up time of 10 years. The cancer cases were rather complete in the diabetes population, as a result of the data linkage with the Dutch National Cancer Registry, where under-registration of cancer was estimated to be lower than 2% [27].

There are also several limitations to be noticed. First, unmeasured lifestyle risk factors such as diet, physical activity, and comorbidity might confound the quality of glycemic control. Second, clinical data including HbA1c were annually collected, whereas the estimate would have been more precise when more HbA1c values within each year were available. Third, this study focused on patients diagnosed with CRC at lower than stage IV, these results cannot be generalized to patients diagnosed with advanced stage CRC. Fourth, as each country has its own diabetes care system, which may be improving over time and defining the quality of glycemic control with different targets values, the generalization of this study is limited to the countries with a primary care system comparable to the Netherlands. Finally, the diabetes management in the ZODIAC database might be influenced by benchmarking. Slightly better quality of glycemic management has been observed in the ZODIAC cohort, as compared to another Dutch outpatient diabetes cohort [28].

## 4. Materials and Methods

### 4.1. Study Design

This prospective cohort study evaluated the quality of glycemic control in the Dutch primary care system, for patients with type 2 diabetes, from 1998 to 2014. Quality of glycemic control for cancer survivors was compared with those with no history of cancer in each follow-up year. The study was reported according to Strengthening the Reporting of Observational Studies in Epidemiology (STROBE) recommendations [29].

### 4.2. Context and Data Source

In the Netherlands, patients with type 2 diabetes were mainly treated in the primary care for their diabetes, according to the national guideline of Dutch College of general practitioners [30].To investigate the management for patients with diabetes treated in the Dutch primary care system, the Zwolle Outpatient Diabetes project Integrating Available Care (ZODIAC) was initiated in 1998. The patients enrolled in the ZODIAC were diagnosed with type 2 diabetes and patients with a short life expectancy, insufficient cognitive abilities, and those being treated in secondary care were excluded from participation. The number of general practitioners who participated increased from 53 since initiation to 731 in 2013, and the number of patients grew from 1622 to 71,648. Each year, each patient received an invitation for an annual check-up, in which an assessment of glycemic control was performed [30]. The quality of diabetes care was benchmarked at general practitioner level each year and had resulted in a complete and good quality of the dataset [22,23,30]. Prospectively collected annual clinical data, including date of diabetes diagnosis, HbA1c values, and medication use since 1998 until the end of 2014, are available.

To obtain cancer-related information including type and date of cancer diagnosis and treatment, the ZODIAC population was linked with the Dutch National Cancer Registry (NCR). The NCR registered more than 98% of cancer cases since the year of 1989 in the Netherlands [31]. Details of the data linkage have been described elsewhere [32]. This data linkage was last updated in December 2020. According to the Dutch Medical Research with Human Subjects Law (Wet Medisch-wetenschappelijk Onderzoek met mensen, WMO), this procedure as well as the data analysis was exempted from formal medical ethics committee review (METC reference number 13.0765).

### 4.3. Study Participants

In this prospective cohort study, we included all patients with type 2 diabetes between January 1998 until December 2014 in the ZODIAC database with clinical follow-up data, who were either a CRC survivor or patients with no cancer history (Figure 1). Patients with lower than stage IV colorectal cancer treated with curative intent in the primary care system were considered to be CRC survivors. Exclusion criteria were (1) a diagnosis of other type of cancer except for non-melanoma skin cancer; (2) a diagnosis of CRC prior to cohort entry; (3) stage IV CRC; (4) rectum cancer treated with radiotherapy only, as these patients were not treated with curative intent based on the Dutch guideline [33]; and (5) less than 1 year clinical follow-up.

### 4.4. Definitions

The baseline year was defined as cohort entry year for all patients. We defined a time-dependent variable indicating the status of CRC. This variable stayed at the status of “no history of cancer” at all follow-up years, as long as there was no diagnosis of CRC, while it switched to the status of ‘diagnosed with CRC’ for all follow-up years that occurred after the CRC diagnosis.

### 4.5. Outcome Measures

The primary outcome was the probability of reaching the HbA1c target level in each follow-up year. The target HbA1c level was defined as ≤53 mmol/mol for patients diagnosed with type 2 diabetes before 2013, based on the 2009 version of the Dutch primary care guideline [34]. As of 2013, this target level for patients aged over 70 years, was loosened according to age and duration of diabetes. For patients diagnosed with type 2 diabetes within 10 years and those who received treatment other than metformin monotherapy, the target was ≤58 mmol/mol. For patients diagnosed with type 2 diabetes for more than 10 years, the target was ≤64 mmol/mol [26]. The target level for these specific patients, therefore, was defined according to the guideline at the time of follow-up. The secondary outcome was the mean of HbA1c in each follow-up year.

### 4.6. Baseline Confounders

Age, gender, duration of diabetes, number of oral glucose-lowering drugs, and use of insulin were considered as baseline confounders because they may differ by cancer status and also influence glycemic control. As the patients entered the cohort in different years and the quality of diabetes care improved over the years [22,23], the baseline calendar year was also included as a confounder in the analysis.

### 4.7. Statistics

Descriptive analyses for baseline characteristics are presented as means with standard deviation for normally distributed values, and median and interquartile range (IQR) for skewed variables. Generalized mixed linear model was used to estimate the probability of reaching the target HbA1c level in each follow-up year and mixed linear model was used to estimate the mean HbA1c during follow-up. For all analyses, the status of cancer was used as a time-dependent variable [35]. In this way, the variable “follow-up year” captured the growth trajectory during the follow-up years for all patients, and the time-varying variable “status of CRC” captured the change in the growth rate that occurred after CRC diagnosis for patients diagnosed with CRC [36,37]. A preliminary inspection of the data, by using “follow-up year” as a categorical variable, revealed no substantial deviation of a linear trajectory of the average growth trajectory, therefore, “follow-up year” was used as a continuous variable. The interaction of “status of CRC” and “follow-up year” captured the annual change rates of the outcomes over years. To account for a patient-specific trajectory, a random intercept and a random slope were allowed in the model with an unstructured covariance matrix. Baseline confounders were adjusted as fixed effects. Assuming that missing data were “missing at random”, the mixed-effects model allowed the use of data for all patients who had at least one year follow-up.

### 4.8. Sensitivity Analysis

To account for possible differences in follow-up duration, sensitivity analyses for the outcomes including only patients being followed for at least 5 years was performed. All statistical tests were two-sided and conducted at the 5% significant level, using STATA/SE 15.0 (StataCorp LLC, College Station, TX, USA) and the SPSS 20.0 software (IBM Corp, Armonk, NY, USA).

## 5. Conclusions

In view of the growing population of cancer survivors who live with concurrent diabetes and have an increased risk of developing cardiovascular diseases, high quality of diabetes care with good glycemic control as a proxy is essential. This prospective cohort study presents comparably high quality of glycemic control for patients with and without CRC in the Dutch primary care, implying a robust diabetes care system and that the diabetes care for CRC survivors can be delegated to primary care professionals.

## Figures and Tables

**Table 1:** Characteristics of patients with type 2 diabetes—diagnosed with colorectal cancer versus no history of cancer.

Characteristics	Colorectal Cancer (*n* = 705)	No Cancer History (*n* = 56,625)	*p*-Values
At cohort entry year			
Age	69.3 ± 8.7	65.0 ± 12.6	<0.001
Male (%)	57.2	50.1	<0.001
Diabetic duration (years)	2.9 (0.8–6.5)	2.5 (0.0–6.1)	0.028
Newly diagnosed with diabetes (%)	28.7	32.5	0.029
HbA1c (mmol/mol) *	49 (43–55)	49 (43–55)	0.514
At target HbA1c (%) *^,†^	75.4	72.2	0.062
Number of oral drugs (%)			
0	15.7	19.6	0.028
1	49.9	50.2	
2	33.8	29.3	
3	0.6	0.9	
Insulin use (%)	0.4	0.9	0.192
During follow-up			
Age at cancer diagnosis	72.7 ± 8.4	n.a.	n.a.
TNM stage (%) ^§^			
In situ	10.8	n.a.	n.a.
I	20.0	n.a.	
II	30.8	n.a.	
III	35.9	n.a.	
Treatment			
Surgery	85.0	n.a.	n.a.
Chemotherapy			
Before surgery	6.4	n.a.	n.a.
After surgery	13.6	n.a.	
Before + after surgery	1.0	n.a.	
Radiotherapy before surgery	18.6	n.a.	n.a.
Number of follow-up years	6 (4–8)	5 (3–7)	<0.001
At least 5 years follow-up (%)	70.5	51.0	<0.001
Number of follow-up years after cancer diagnosis	2 (1–4)	n.a.	n.a.

Normally distributed variables presented as mean SD. Non-normally distributed data presented as median (IQR). * When the measurement in the baseline year was missing, the first available measurement was used. ^†^ The target HbA1c level was defined as ≤53 mmol/mol for patients diagnosed with type 2 diabetes before 2013, based on the 2009 version of the Dutch primary care guideline. As of 2013, this target level for patients aged over 70 years was loosened, according to age and duration of diabetes. For patients with less than 10 year duration of diabetes who received treatment other than metformin monotherapy, the target was ≤58 mmol/mol. For patients with more than 10 year duration of diabetes, the target was ≤64 mmol/mol. The target level for these specific patients, therefore, was defined according to the guideline at the time of follow-up. ^§^ A total of 2.6% of the TNM stages were unknown. n.a.: Not applicable.

**Table 2:** Estimated quality of glycemic control during follow–up.

Follow-Up	Colorectal Cancer (*n* = 705)			No Cancer History (*n* = 56,625)		
	At Target level % (95% CI)	Mean HbA1c mmol/mol (95% CI)	Number of Patients with Available HbA1c Data (*n*)	At Target Level % (95% CI)	Mean HbA1c mmol/mol (95% CI)	Number of Patients with Available HbA1c Data (*n*)
Year 0 (Baseline *)	0.75 (0.70–0.80)	49.1 (47.6–50.5)	111	0.75 (0.75–0.76)	49.9 (49.9–50.0)	56,256
Year 1	0.76 (0.72–0.80)	49.2 (48.2–50.1)	225	0.76 (0.75–0.76)	50.2 (50.1–50.2)	56,142
Year 2	0.77 (0.74–0.80)	49.3 (48.5–50.1)	316	0.76 (0.75–0.76)	50.4 (50.3–50.5)	56,050
Year 3	0.76 (0.73–0.79)	50.0 (49.3–50.7)	345	0.75 (0.75–0.75)	50.7 (50.6–50.7)	45,575
Year 4	0.75 (0.72–0.78)	50.5 (49.8–51.2)	354	0.74 (0.73–0.74)	51.0 (50.9–51.0)	37,514
Year 5	0.74 (0.71–0.77)	51.2 (50.5–51.9)	361	0.72 (0.72–0.73)	51.3 (51.3–51.4)	31,910
Year 6	0.72 (0.69–0.76)	51.9 (51.0–52.7)	299	0.70 (0.70–0.71)	51.7 (51.6–51.8)	22,032
Year 7	0.70 (0.66–0.74)	52.9 (51.9–53.8)	277	0.69 (0.69–0.69)	52.1 (52.0–52.2)	17,468
Year 8	0.67 (0.63–0.72)	53.6 (52.5–54.7)	213	0.68 (0.67–0.68)	52.4 (52.2–52.5)	12,035
Year 9	0.66 (0.59–0.73)	54.0 (52.0–55.9)	92	0.64 (0.63–0.65)	52.9 (52.7–53.2)	5698
Year 10	0.66 (0.59–0.74)	54.1 (52.0–56.3)	79	0.63 (0.62–0.64)	53.0 (52.8–53.3)	4548

The number of CRC survivors first increased as a result of developing CRC during follow-up, and then decreased because of reaching the end of follow-up. All analyses were corrected for baseline confounders including age, gender, duration of diabetes, number of oral drugs, insulin use, and baseline year. * Baseline year was defined as cohort entry year.

## Data Availability

All data are available upon request from the corresponding author.

## Supplementary Materials

The following are available online at https://www.mdpi.com/article/10.3390/cancers13112767/s1, Figure S1: Estimated mean of the outcomes in sensitivity analysis (A): Estimated mean probability of at target HbA1c level during follow-up among patients with at least 5 years follow-up. (B): Estimated mean HbA1c (mmol/mol) during follow-up among patients with at least 5 years follow-up, Table S1: Parameter estimates of the fixed effects for the mixed model analysis of the change of probability at target HbA1c, Table S2: Parameter estimates of the fixed effects for the mixed model analysis of the change in mean HbA1c, Table S3: Parameter estimates of the fixed effects for the mixed model analysis of the change of probability at target HbA1c among patients being followed for at least 5 years, Table S4: Parameter estimates of the fixed effects for the mixed model analysis of the change in mean HbA1c among patients being followed for at least 5 years.
