# Supplementary material for: Glycemic Control for Colorectal Cancer Survivors Compared to Those without Cancer in the Dutch Primary Care for Type 2 Diabetes: A Prospective Cohort Study
Source: Cancers (Basel). 2021 Jun 2;13(11):2767. doi: 10.3390/cancers13112767 (PMC8199666; doi:10.3390/cancers13112767)
Supplement: Supplementary file 1 [file cancers-13-02767-s001.zip › cancers-1219031 Supplementary final update.pdf]

cancers

MDPI

Supplementary Material: Glycemic Control for Colorectal Cancer Survivors Compared to Those Without Cancer in the Dutch Primary Care for Type 2 Diabetes: A Prospective Cohort Study

Jing de Haan-Du, Gijs W. D. Landman, Nanne Kleefstra, Dennis Schrijnders, Marjolijn Manders, Amanda C. R. K. Bos, Cathrien Tromp-van Driel, Petra Denig, Klaas H. Groenier and Geertruida H. de Bock

A

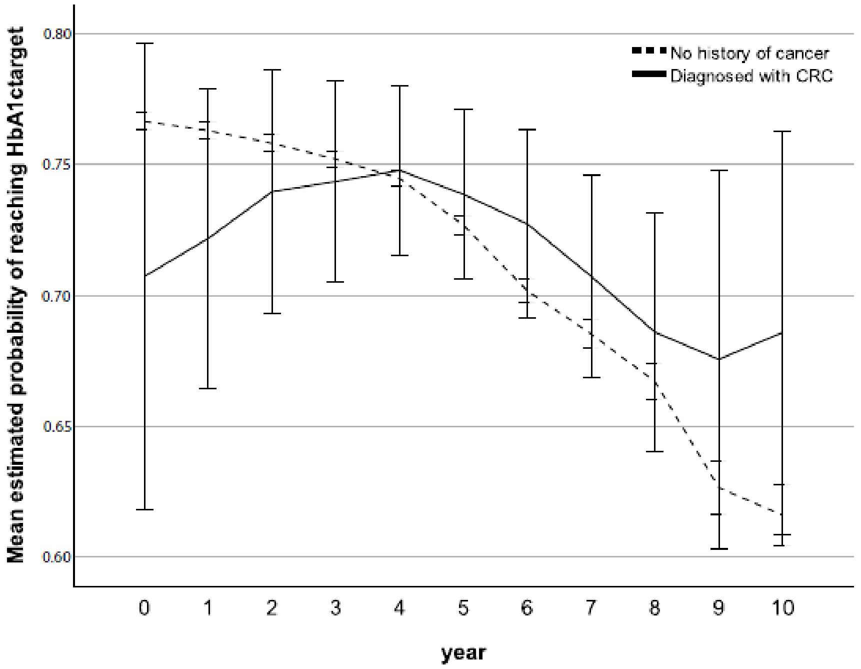

B

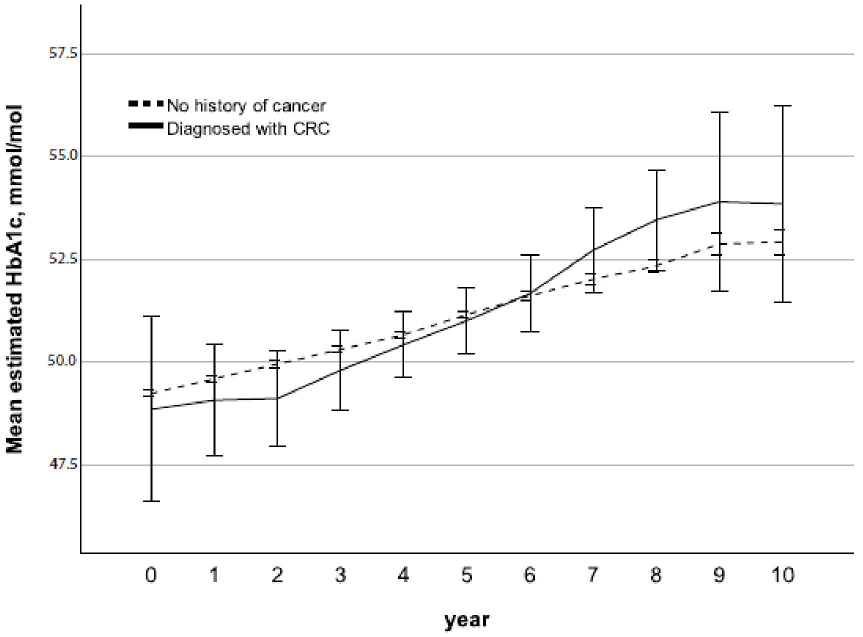

Figure S1. Estimated mean of the outcomes in sensitivity analysis. (**A**) Estimated mean probability of at target HbA1c level during follow-up among patients with at least 5 years follow-up. (**B**) Estimated mean HbA1c (mmol/mol) during follow-up among patients with at least 5 years follow-up. Year 0 represents the cohort entry year, and year 10 represents the data for patients being followed for 10 years. Adjusted for baseline age, gender, diabetes duration, number of oral drugs, use of insulin and baseline year. The horizontal bars represent for the 95% confidence intervals. Abbreviation: HbA1c, glycated hemoglobin.

Table S1. Parameter estimates of the fixed effects for the mixed model analysis of the change of probability at target HbA1c.

Parameter	Estimate coefficient	*p*-Value	95% Confidence Interval	
			Lower Bound	Upper Bound
Intercept	−52.52	<0.001	−68.91	−36.13
Follow-up year	−0.02	0.001	−0.02	−0.01
No history of cancer status	Reference	.	.	.
Colorectal cancer status	−0.28	0.157	−0.66	0.11
No history of cancer status*Follow-up year	Reference	.	.	.
Colorectal cancer status*Follow-up year	0.05	0.260	−0.03	0.12
Male	Reference	.	.	.
Female	0.07	0.004	0.02	0.12
Age at baseline	0.03	<0.001	0.03	0.03
Duration of diabetes at baseline	−0.09	<0.001	−0.10	−0.09
Baseline year	0.03	<0.001	0.02	0.04
Number of oral drugs at baseline	−1.28	<0.001	−1.32	−1.24
Insulin use at baseline	−2.13	<0.001	−2.13	−1.88

Table S2. Parameter estimates of the fixed effects for the mixed model analysis of the change in HbA1c.

Parameter	Estimated Coefficient	*p*-Value	95% Confidence Interval	
			Lower Bound	Upper Bound
Intercept	−78.77	0.001	−125.70	−31.83
Follow-up year	0.23	<0.001	0.21	0.25
No history of cancer status	Reference	.	.	.
Colorectal cancer status	−0.91	0.045	−1.80	−0.02
No history of cancer status*Follow-up year	Reference	.	.	.
Colorectal cancer status*Follow-up year	0.30	0.001	0.12	0.48
Male	Reference	.	.	.
Female	−0.09	0.172	−0.23	0.04
Age at baseline	−0.07	<0.001	−0.07	−0.06
Duration of diabetes at baseline	0.27	<0.001	0.26	0.29
Baseline year	0.06	<0.001	0.04	0.09
Number of oral drugs at baseline	3.49	<0.001	3.39	3.59
Insulin use at baseline	6.85	<0.001	6.11	7.59

Table S3. Parameter estimates of the fixed effects for the mixed model analysis of the change of probability at target HbA1c among patients being followed for at least 5 years.

Parameter	Estimated Coefficient	*p*-Value	95% Confidence Interval	
			Lower Bound	Upper Bound
Intercept	−77.61	<0.001	−100.42	−54.81
Follow-up year	−0.05	<0.001	−0.06	−0.04
No history of cancer status	Reference	.	.	.
Colorectal cancer status	−0.59	0.018	−1.08	−1.02
No history of cancer status*Follow-up year	Reference	.	.	.
Colorectal cancer status*Follow-up year	0.10	0.032	0.01	0.19
Male	Reference	.	.	.
Female	0.08	0.006	0.02	0.14
Age at baseline	0.03	<0.001	0.03	0.03
Duration of diabetes at baseline	−0.09	<0.001	−0.10	−0.08
Baseline year	0.04	<0.001	0.03	0.05
Number of oral drugs at baseline	−1.21	<0.001	−1.26	−1.16

Insulin use at baseline	−1.94	<0.001	−2.22	−1.67

Table S4. Parameter estimates of the fixed effects for the mixed model analysis of the change in mean HbA1c among patients being followed for at least 5 years.

Parameter	Estimated Coefficient	*p*-Value	95% Confidence Interval	
			Lower Bound	Upper Bound
Intercept	75.06	0.025	9.64	140.47
Follow-up year	0.36	<0.001	0.34	0.38
No history of cancer status	Reference	.	.	.
Colorectal cancer status	−0.61	0.283	−1.72	0.50
No history of cancer status*Follow-up year	Reference	.	.	.
Colorectal cancer status*Follow-up year	0.24	0.019	0.04	0.44
Male	Reference	.	.	.
Female	−0.04	0.650	−0.21	0.13
Age at baseline	−0.07	<0.001	−0.07	−0.06
Duration of diabetes at baseline	0.26	<0.001	0.24	0.27
Baseline year	−0.01	0.428	−0.05	0.02
Number of oral drugs at baseline	3.18	<0.001	3.05	3.31
Insulin use at baseline	6.01	<0.001	5.18	6.85

Publisher's Note: MDPI stays neutral with regard to jurisdictional claims in published maps and institutional affiliations.

© 2021 by the authors. Licensee MDPI, Basel, Switzerland. This article is an open access article distributed under the terms and conditions of the Creative Commons Attribution (CC BY) license (http://creativecommons.org/licenses/by/4.0/).
